# Anti‐cancer bioactivity of sweet basil leaf derived extracellular vesicles on pancreatic cancer cells

**DOI:** 10.1002/jex2.142

**Published:** 2024-02-01

**Authors:** Uday Chintapula, Daniel Oh, Cristina Perez, Sachin Davis, Jina Ko

**Affiliations:** ^1^ Department of Pathology and Laboratory Medicine, Perelman School of Medicine University of Pennsylvania Philadelphia Pennsylvania USA; ^2^ Department of Bioengineering, School of Engineering and Applied Sciences University of Pennsylvania Philadelphia Pennsylvania USA

**Keywords:** anti‐cancer, basil plant, extracellular vesicles, pancreatic cancer, therapeutics

## Abstract

Most living organisms secrete tiny lipid bilayer particles encapsulating various biomolecular entities, including nucleic acids and proteins. These secreted extracellular vesicles (EVs) are shown to aid in communication between cells and their environment. EVs are mainly involved in the signalling and manipulation of physiological processes. Plant EVs display similar functional activity as seen in mammalian EVs. Medicinal plants have many bioactive constituents with potential applications in cancer treatment. Particularly, Basil (*Ocimum basilicum*), has wide therapeutic properties including anti‐inflammatory, anti‐cancer, and anti‐infection, among others. In this study, we focused on using EVs purified from Apoplast Washing Fluid (AWF) of Basil plant leaves as a biological therapeutic agent against cancer. Characterization of Basil EVs revealed a size range of 100–250 nm, which were later assessed for their cell uptake and apoptosis inducing abilities in pancreatic cancer cells. Basil plant EVs (BasEVs) showed a significant cytotoxic effect on pancreatic cancer cell line MIA PaCa‐2 at a concentration of 80 and 160 μg/mL in cell viability, as well as clonogenic assays. Similarly, RT‐PCR and western blot analysis has shown up regulation in apoptotic gene and protein expression of Bax, respectively, in BasEV treatment groups compared to untreated controls of MIA PaCa‐2. Overall, our results suggest that EVs from basil plants have potent anti‐cancer effects in pancreatic cancer cells and can serve as a drug delivery system, demanding an investigation into the therapeutic potential of other medicinal plant EVs.

## Graphical abstract

Basil plant EVs extracted from basil plant inhibit pancreatic cancer cell growth *in vitro*.

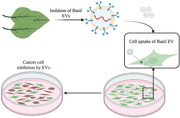

## 1 INTRODUCTION

Cancer is a public health problem with various treatment strategies being employed including chemotherapy, radiotherapy, immunotherapy, and surgery (Debela et al., 2021; Naran et al., 2018; Siegel et al., 2021). Despite various innovations in cancer therapy, the treatment strategies still suffer from poor targeting, limited scale‐up capability, and low biocompatibility, leading to toxic side effects (Timmer et al., 2021). Pancreatic cancer in specific is the fourth leading cause of cancer death and with decades of research, the 5‐year survival rate remains very low (Li et al., 2022). The use of novel drug delivery systems like nanoparticles or even cell‐loaded nanoparticles is employed to improve drug accumulation at tumour sites (Cheng et al., 2021; Liu et al., 2017). With minimal success, these strategies still face the challenges of physiological barriers from efficient cellular delivery and to avoid clearance by the immune system. Hence, alternative treatment strategies are needed to address the drawbacks of current therapies.

Cell‐secreted EVs are small 50–1000 nm lipid bilayer particles that show superior ability in transferring biological agents to cells compared to nanoparticles. This superiority is mostly due to their intrinsic lipid rafts and structural protein abilities to evade the immune response, improving overall accumulation in tumours via Enhanced Permeability and Retention (EPR) effects, and anti‐cancer efficacy of the drug delivered (Herrmann et al., 2021). Recent reports demonstrate the ability to load cargo including proteins, nucleic acids, and chemotherapy drugs into EVs for drug delivery and gene therapy (Cabeza et al., 2020). Despite the advancement in mammalian EV engineering for drug delivery, isolation standards, and clinical grade purification are lacking, along with scalable manufacturing of well‐characterized EVs (Pirisinu et al., 2022). The yield of EVs using current isolation techniques needs to be drastically improved to achieve scalability for developing treatments.

Clinical grade purity needs to be achieved as EVs isolated from different subjects or samples are prone to carryover unspecific EVs like apoptotic bodies, exomeres, supermeres, microvesicles, and other cell debris which can cause adverse reactions when administered. EV characterization including its external lipid‐protein, internal nucleic acids, and other biologicals, is still an evolving field. Overall, significant challenges still need to be addressed before the application of mammalian EVs for cancer treatments.

First observed in the ‘60s, plant‐derived extracellular vesicles (PDEV) have been overlooked until the recent decade (Halperin, 1969). Similar to mammalian cell‐derived EVs, PDEVs are involved in signalling such as the formation of a cell wall, plant defence against fungi, microbe interactions, and other unexplored bioactivities (Bleackley et al., 2020; Chukhchin et al., 2019; He et al., 2021; Regente et al., 2017; Rutter & Innes, 2018). PDEVs have been shown to transfer small non‐coding RNAs, indicating their ability for gene regulation comparable to mammalian exosomes (Cui et al., 2020; Urzì et al., 2021). With potent bioactivity and the ability to transfer biological agents across cells, PDEVs serve as a promising new therapeutic agent as well as a drug delivery system (Dad et al., 2021). With high scalability in EV isolation from plants and numerous varieties of plants readily available, PDEVs provide an exciting solution in addressing challenges with the use of mammalian EVs for therapy.

Here, we explored PDEVs from the Basil plant whose extracts were shown to possess biological activity against pancreatic cancer cells (Shimizu et al., 2013). Basil plant EVs (BasEVs) were extracted via infiltration centrifugation from basil leaves and initial characterization displayed similar physical characteristics of animal EVs. We investigated the apoptotic effects of BasEVs on pancreatic cancer cells using various *in vitro* assays. The results show a biotechnological approach for exploring the potency of other high medicinal value plants for their pro‐apoptotic activity against cancer cells.

## 2 RESULTS

### 2.1 BasEV isolation and characterization

**FIGURE 1:**
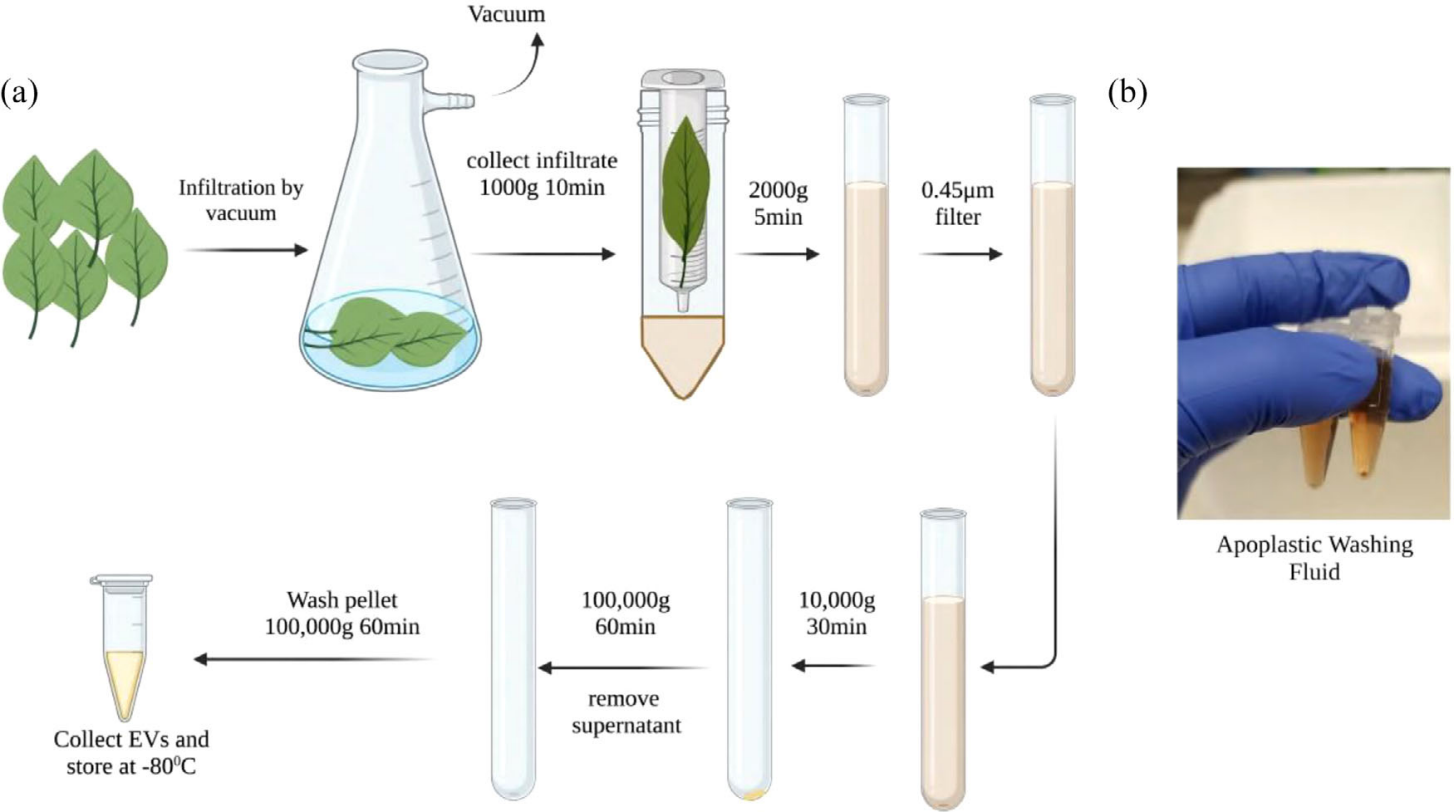
(a) Schematic representation of BasEV isolation from Basil plant leaves. (b) Apoplast Washing Fluid (AWF) collected from basil leaves.

**FIGURE 2:**
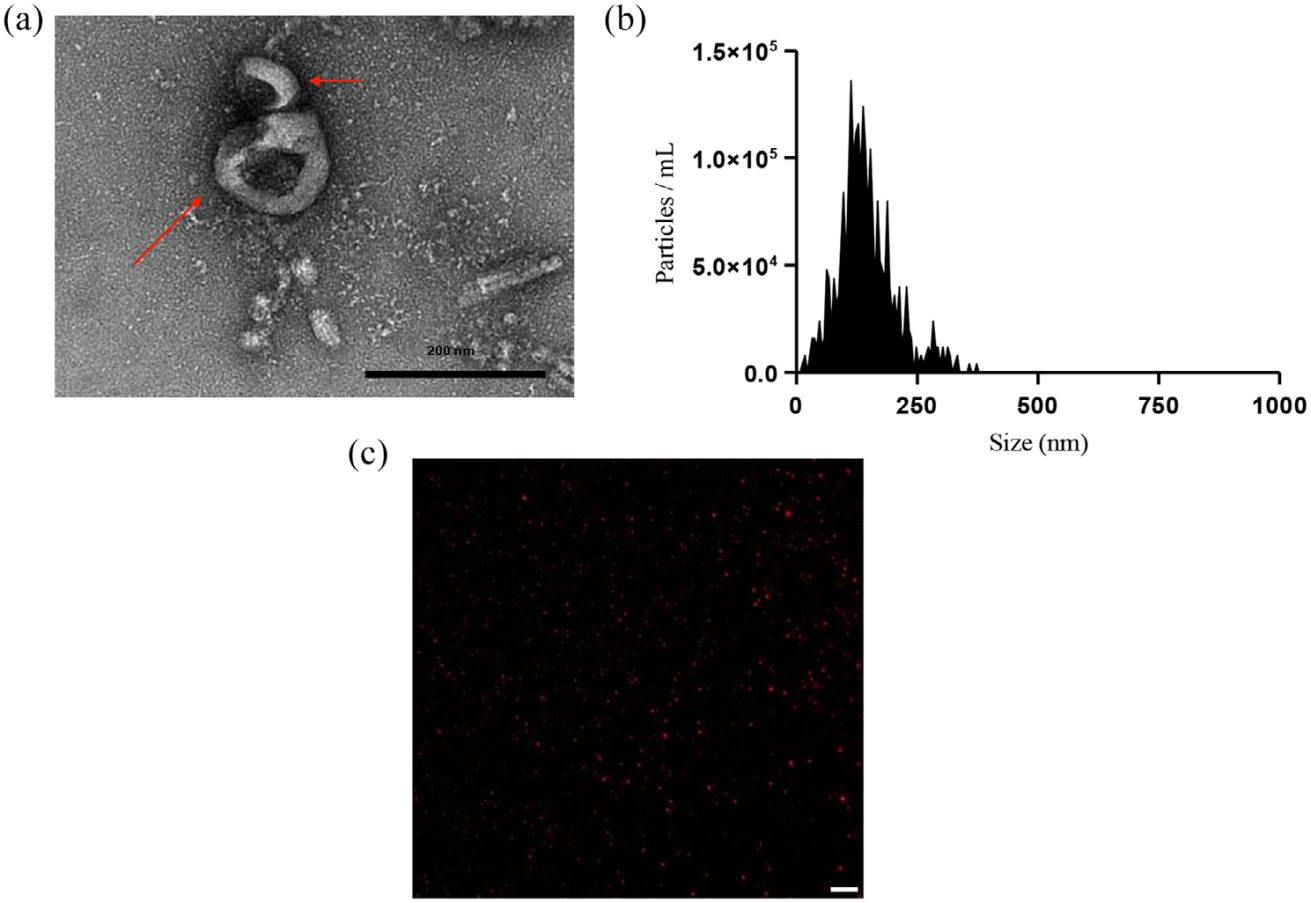
Characterization of BasEVs. (a) TEM image of BasEVs with red arrows pointing to BasEVs. (b) NTA analysis of BasEV concentration on Y‐axis and size represented on X‐axis, with peak size at 138 nm. (c) DiI stained BasEVs in TRITC channel of a fluorescent microscope indicate intact lipid bilayer in Isolated EVs, Scale bar‐20 μm.

Fresh Basil plants were used as a source for EV isolation. Leaves cut from the plant were washed and vacuum‐in‐filtered with infiltration buffer (Figure 1a). Apoplastic washing fluid from Basil Plant EVs show brown coloured suspension indicating the presence of extract from leaves in the infiltration buffer (Figure 1b). Leaves with proper infiltration turned dark green, compared to the light green of the leaves with air still present in between the cell walls. To yield 200–500 μg of BasEVs, 200–500 g of leaves were required (Table S1). TEM images reveal the spherical morphology of EVs with a size ranging between 50 and 150 nm (Figure 2a). Nanoparticle Tracking Analysis (NTA) revealed that the EV sizes ranged from 100 to 250 nm with a major peak at 135 nm (Figure 2b). EV concentration determined by NTA was 3–4.5 × 10^10^ EVs/mL for leaves weighing 200–500 g. We also observed that 85% of EVs were in the range of 100–150 nm with a small percentage (<6%) in the high range of 290 nm, probably generated from the aggregation of EVs. Smaller EVs were also seen with <9% population reported by NTA (Figure S1). To visualize the BasEVs, DiI lipophilic dye staining was applied and later observed via fluorescence imaging of BasEVs, which indicated the presence of lipids (Figure 2c). Heat shock proteins (HSP), especially HSP70 and GAPDH which are conserved across species, were used to stain proteins in basil EV and mammalian EVs (A431 lung cancer cell). Mammalian cell EVs had a strong signal for HSP70 and GAPDH while BasEVs shows a weak signal of HSP70 and no signal in GAPDH (Figure S2).

### 2.2 Internalization of BasEVs in human pancreatic cancer cell lines

**FIGURE 3:**
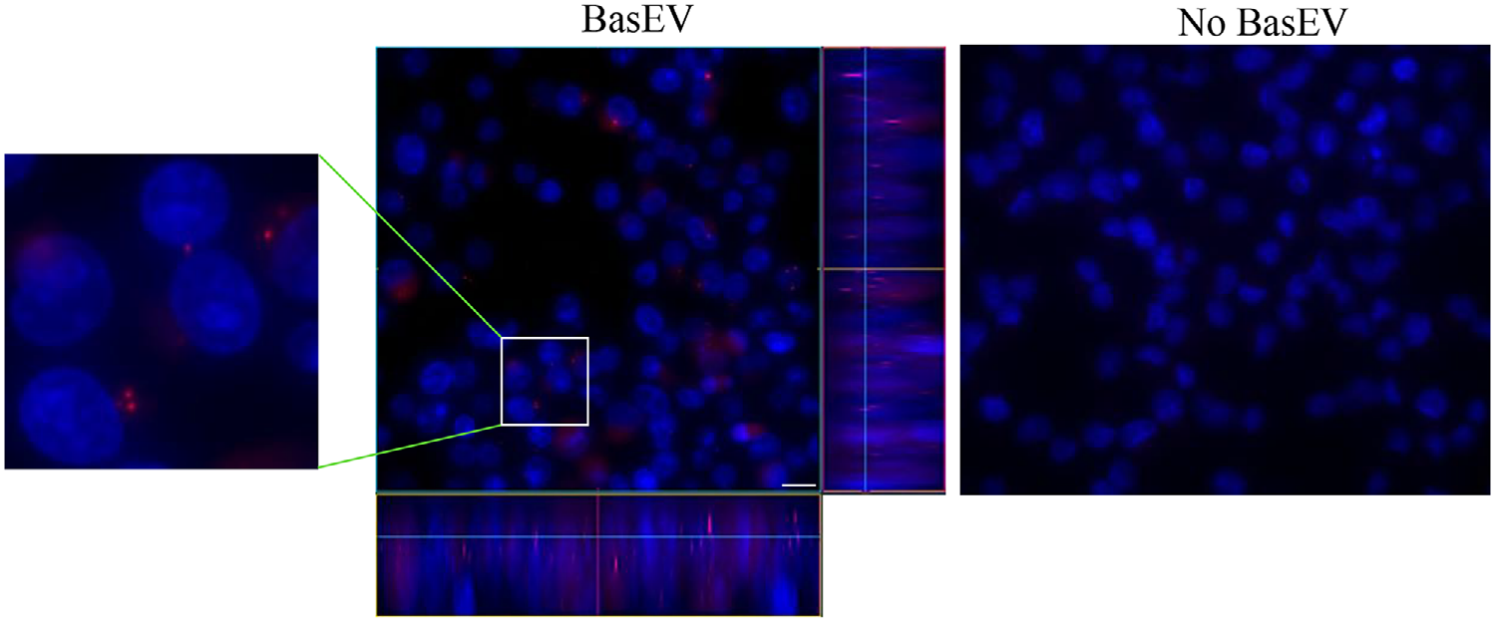
BasEVs uptake in pancreatic cancer cells. Colour stacked images showing cell contour, nuclei (blue) and BasEV (red) in Mia PaCa‐2 cells treated with BasEVs (middle) and no BasEVs (right). A zoomed in ROI (on left) showing BasEV localization in cells. (middle) Z‐stacked image showing colocalization of BasEV and nucleus represented on the Z projection on right and below the BasEV treated cell image. Scale bar is 20 μm.

After characterizing the BasEVs, we further explored their uptake in pancreatic cancer MIA PaCa‐2 cells. BasEVs incubated with pancreatic cancer cells showed uptake ability within 4 h. Here, cells were stained with Hoechst stain to capture z‐stack images showing uptake of DiI‐stained BasEVs in the cells (Figure 3). After 4 h, BasEVs uptake was observed inside the cytoplasm with very few seen around the cell membrane. A z‐stack image of MIA PaCa‐2 cell lines shows distinct punctates of BasEV inside the cytoplasm with uniform distribution (Video S1).

Similarly, when BasEVs were incubated with other pancreatic cancer cell lines and a similar uptake was observed in both AsPC‐1 and PanC1 cell lines (Figure S3).

### 2.3 Biological effects of BasEVs on MIA PaCa‐2 pancreatic cancer cells

**FIGURE 4:**
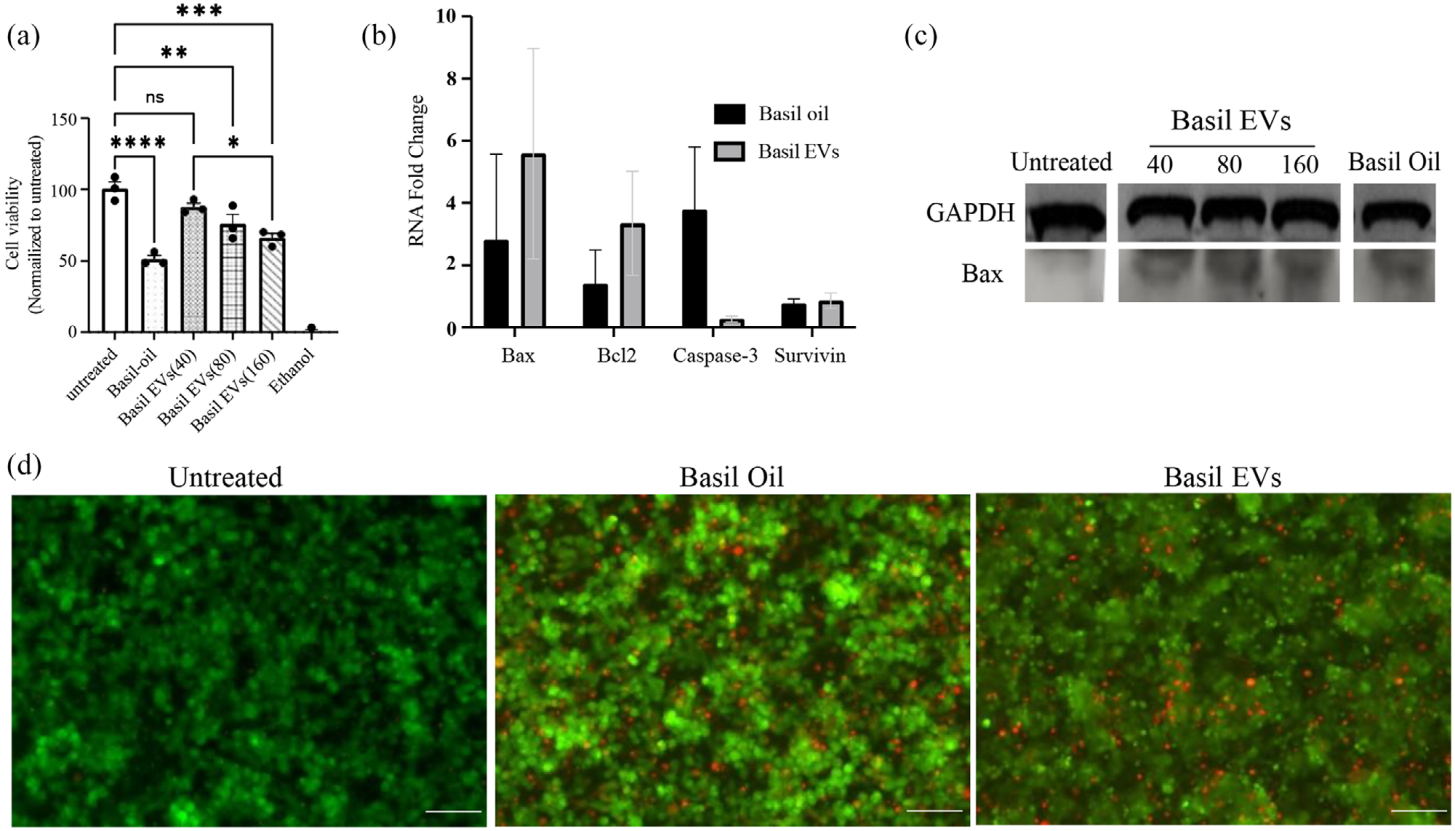
BasEVs inhibit cell growth in MIA PaCa‐2 cells. (a) MTS assay results showing a dose‐ dependent reduction in cell viability of MIA PaCa‐2 cells after BasEV treatment. (b) RT‐PCR analysis of apoptosis‐relevant mRNA expression levels upregulated upon treatment with BasEV. (c) Western blot of Bax and GAPDH protein levels in Mia PaCa‐2 treated with various concentrations of BasEVs (40, 80, and 160 μg/mL). (d) LIVE/DEAD assay images of MIA PaCa‐2 cells showing propidium iodide‐stained dead cells and calcein green stained live cells. Scale bar is 260 μm. Standard error mean was used to plot the graphs with *P ≤ 0.05, **P ≤ 0.01, ***P ≤ 0.001, and ****P ≤ 0.0001.

Recently, plant EVs have been rediscovered for their therapeutic properties for cancer treatments (Boccia et al., 2022; Nemati et al., 2022; Tan et al., 2022). With studies showing Basil plant extract as a treatment for pancreatic cancer, we explored the anti‐cancer effects of other biological entities of the Basil plant, such as their EVs (Sud et al., 2017). BasEVs given at various concentrations (0–160 μg/mL) to MIA PaCa‐2 pancreatic cells showed inhibitory effects on cancer cell proliferation (Figure 4a) while similar concentrations did not have any significant effect on HEK293T cells (Figure S4). With basil oil extract as a positive control (IC50 at 1.7% v/v), BasEVs showed inhibition of pancreatic cancer cell growth at 80 and 160 μg/mL. BasEVs exhibited a dose‐dependent inhibition of pancreatic cancer cells with a lower concentration of 40 μg/mL having no significant effect compared to untreated cells.

Higher concentration of 160 μg/mL showed no significance compared to IC50 of basil oil extracts. A LIVE/DEAD assay was performed showing increased cell death from the treatment of BasEVs in MIA PaCa‐2 pancreatic cancer cells comparable to basil oil extracts treatment (Figure 4d). Furthermore, clonogenic survival assay further supports the inhibitory effects of BasEVs in MIA PaCa‐2 cells, with reduction in number of colonies formed after treatment with BasEVs (Figure S5).

### 2.4 Upregulation of apoptotic activity after BasEVs treatment

To further assess the biological effects of BasEVs, we performed an RT‐PCR on the RNA extracted from the MIA PaCa‐2 pancreatic cancer cell lines after BasEV treatment (primers used presented in Table S2). A mix of apoptotic (Bax, Caspase‐3), Bcl2 (anti‐apoptotic) and metastatic (Survivin) related genes were studied via RT‐PCR. BasEVs showed a ∼5‐fold increase in pro‐apoptotic Bax gene expression, also higher than the positive control (Figure 4b). Mean Bax/Bcl2 ratio for basil oil and BasEVs were 2 and 1.6, respectively, indicating of pro‐apoptotic activity. The caspase‐3 gene expression was up regulated with ∼4‐fold increase in Basil oil treatment group compared negligible increase in BasEV treated cells. There was no significant up regulation seen in pro‐metastatic survivin genes in BasEVs or positive control. Western blot analysis of BasEV treated Mia PaCa‐2 cell lysate proteins showed an increased Bax protein expression compared to the untreated controls (Figure 4c). Increase in protein band intensities were observed with increasing concentrations. Overall, BasEVs showed a significant fold increase in pro‐apoptotic markers compared to the untreated group and pro‐apoptotic gene expression higher than basil oil positive control.

Additionally, we performed an experiment to assess combinatorial effects of BasEVs and their synergy with gemcitabine chemo drug, commonly used in pancreatic cancer treatments. Our results show that there is significant reduction in cell viability when BasEVs and gemcitabine are co‐administered to the cells rather than BasEVs pretreatment followed by gemcitabine delivery or individual component treatments (Figure S6). Caspase activity assay performed on MIA PaCa‐2 cells treated with both BasEVs, and basil oil had increased activity compared to untreated control but no significance between BasEVs and basil oil groups was observed (Figure S7).

### 2.5 Proteomic analysis of BasEVs

We assessed the effects of BasEVs and the supernatant from BasEV isolation by normalizing their protein concentrations. With an exposure for 16 h, we observed that the supernatant had an inhibitory effect on MIA PaCa‐2 cells comparable to BasEVs indicating further assessment of protein contents (Figure S8). Towards that extent, we performed the proteomic analysis of BasEVs and the supernatants. Using tryptic digestion of BasEV pellets and supernatant consisting of apoplastic washing fluid, a total of 124 individual proteins were revealed when compared with database of *Oscimum basilicum* species (basil plant) (Figure S9). Of the 124 proteins identified, we were able to shortlist unique proteins and their functions (Table S3). BasEV proteins showed functions associated with cellular metabolism such as photosynthesis and other regulatory mechanisms. Sweet basil produces aromatic eugenol and chavicol as the major phenylpropenes and molecules with anti‐cancer properties (Dhar et al., 2020; Koeduka et al., 2006). Evidently, we observed eugenol (potent anti‐ cancer agent) synthesis related enzymes of Eugenol O‐methyltransferase, which was mainly detected in BasEVs alone, while eugenol synthase was present in both BasEVs and apoplastic washing fluid supernatants.

## 3 DISCUSSION

Sweet basil (*O. Basilicum*) plant of the mint family has shown tremendous pharmaceutical benefits with its leaves being used in Indian traditional medicine as an ailment for fevers, flu, asthma, bronchitis, influenza, and diarrhoea (Shahrajabian et al., 2020). Exploring Basil, specifically for cancer treatments, led to studying the effects of their extracts on pancreatic cancer, which showed detrimental effects on pancreatic cell proliferation and viability. EVs extracted from leaf apoplast carry stress‐response proteins as revealed by proteomic studies (Rutter & Innes, 2018). With presence of internal active biological entities, plant EVs may hold therapeutic potential against various diseases owing to their cross‐kingdom RNA interference and other defence mechanisms (Cai et al., 2018; He et al., 2021; Regente et al., 2017).

Various proteins involved in reactive oxygen species signalling and membrane trafficking have been observed in the EV proteome. Proteins such as PEN1, SYP122, and SYP132 observed in EV proteome have previously been shown to contribute towards generating immune responses (Rutter & Innes, 2017). As evident from previous studies, proteins are protected inside the EV lumen, and uptake of these proteins via EVs may have detrimental effects in cells (Rutter & Innes, 2017). Toward that approach, here we explored BasEVs for their biological activity against pancreatic cancer, with an interest in developing a drug carrier for combinatorial treatments (Wang et al., 2020). Although use of plant EVs for drug delivery is highly scalable, loading of exogenous cargo into plant EVs can have its challenges including low exogenous cargo loading capacity, unknown uptake activity across species, and pre‐existing unknown bioactive compounds, causing unfavourable side effects. Screening of plant EVs using a systematic approach performed in this research can aid in identifying other promising plant EV candidates for drug delivery.

BasEVs were isolated directly from AWF of sweet basil plant leaves excluding cytoplasmic components, hence improving the purity compared to other techniques where crushing or breaking down of structures lead to contaminations from chloroplasts, cell debris, etc. (Liu et al., 2020). Along with exclusion of cytoplasmic contaminants, other soluble protein and chemical components are also excluded with the ultra‐high‐speed collection of EV pellets isolated and enriched in the last two steps of synthesis. Isolated BasEVs showed sizes of 100–250 nm comparable to other studies involving isolation from apoplast washing fluid, for example, *Arabidopsis thaliana* leaves, with PDEVs slightly larger than the prominent exosome mammalian EVs ranging between 30 and 150 nm (Huang et al., 2021; Liu et al., 2020; Szatanek et al., 2015; Zand Karimi et al., 2022). TEM images reveal a spherical bubbles or cups resembling EVs with an endocytic origin. BasEVs visualized with a fluorescent microscope had a spherical morphology under lipophilic dye staining, indicating the presence of lipids such as lipid bilayers observed in typical EV structures. Although, an appreciable signal from HSP70 antibodies was seen in BasEVs western blot, a more robust antibodies need to be developed to target plant‐based proteins for BasEV protein characterization. Taken together, these data indicate the presence of extracellular vesicles isolated from AWF of basil plant leaves via the infiltration centrifugation method.

BasEVs showed uptake in the MIA PaCa‐2 cell line within 4 h of treatment, with evidence of their encapsulation inside the cytoplasm. Similar to our results, previous reports indicate the ability of mammalian cells to uptake PDEVs (Ly et al., 2023; Timmer et al., 2021). Other pancreatic cells including AsPC‐1 and PanC1 also showed similar uptake ability, but BasEVs uptake in other cancer cell lines along with their uptake mechanisms is yet to be determined. A dose‐dependent inhibition in MIA PaCa‐2 cell viability was observed indicating the potent activity of BasEVs. BasEVs cancer cell inhibition at 160 μg/mL is comparable to IC50 of basil oil extract seen in pancreatic cancer cells, insinuating potency of EVs comparable with phytochemicals present in oil extracts (Shimizu et al., 2013). In a similar study, Boccia et al. showed that *salvia dominica* plant hairy root derived EVs show selective potency towards pancreatic and mammary cancer cells (Boccia et al., 2022). While BasEVs showed reduction in viability of pancreatic cancer cell lines, similar treatment of BasEVs on host cells of HEK293T did not show any significant difference compared to untreated control. Additionally, supporting BasEV potent bioactivity in cancer cells, their treatment showed a reduced clonogenic survival in MIA PaCa‐2 cells compared to untreated cells. We further investigated if the AWF supernatants show similar inhibitory properties such as BasEVs. Upon treatment with supernatants from ultracentrifugation (100,000×*g*) spins, Mia PaCa‐2 cells showed similar cytotoxicity comparable to BasEVs. The inhibitory properties can be explained by the various small chemical molecules (e.g., eugenol, chavicol and other polyphenols) that may be present in the supernatants as well as the infiltration buffer carried over in the supernatants.

RT‐PCR results demonstrate BasEVs apoptosis triggering ability with the elevated expression of apoptotic Bax protein in on BasEV treated MIA PaCa‐2 cells. To note, the caspase‐3 apoptotic gene or pro‐metastatic survivin genes were of no significant change in both western blot and caspase activity assay performed on BasEV treated cells, indicating other mechanisms of apoptosis driven by BasEVs, requiring further investigation to understand their apoptotic activity. Bax protein expression may enhance the activity of chemo drugs including gemcitabine and 5‐Fu, as reported previously (Xu et al., 2002). With data showing increase in cell Bax protein expression on treatment with BasEVs, a combinatorial treatment of BasEV and chemo drugs can be developed either by loading chemo drugs into EVs or a sequential treatment with BasEVs followed by chemo drugs. Towards that approach, our initial assessment does show a combinatorial inhibitory effect in MIA PaCa‐2, with significant inhibition in the presence of BasEVs and gemcitabine. Interestingly, pretreatment of BasEVs 4 h prior to the gemcitabine chemo drug treatment did not see any significant cell inhibition, indicating co‐administration has no antagonistic effects. To the best of our knowledge, this is the first time EV derived from sweet basil plant leaves is shown to be uptaken by mammalian cells and display potent activity against cancer.

Peptide extracts from EV pellets and AWF supernatants were analysed using a mass spectrometer and various proteins identified. Among these proteins, we identified eugenol O‐ methyltransferase and eugenol synthase in EV population which may be involved in production of eugenols (potent anti‐cancer agent) from available phenylpropenes, coniferyl alcohols, or other esters (Al‐Sharif et al., 2013; Jaganathan & Supriyanto, 2012; Koeduka et al., 2006; Padhy et al., 2022; Zari et al., 2021). Eugenol has previously shown apoptotic properties as well as synergy with cisplatin in cancer cells (Al‐Sharif et al., 2013; Fathy et al., 2019; Wijewantha et al., 2023). Study of chemical components in these EVs can help identify other substrate (e.g., ester) molecules that can be used by these eugenol synthesis enzymes detected in BasEVs (Koeduka et al., 2006). Further studies involving more advanced database search and use of other analytical techniques (e.g., metabolomics) can outline complete list of BasEV components that may be involved in the apoptotic effects observed in BasEV treatment of pancreatic cancer cells.

Basil extracts such as polyphenols, flavonoids, and other phytochemicals have already displayed anti‐tumorigenic effects on different cancers (Dhar et al., 2020; Koeduka et al., 2006; Perna et al., 2022). However, the ability of EVs to be loaded with chemo drugs and accommodate surface modifications can enable combinatorial treatment approaches with targeted drug delivery to achieve higher therapeutic efficacy. Some examples showing plant‐derived EV for drug delivery application include the use of grapefruit EVs surface functionalized with aptamer loaded with doxorubicin showing successful targeting against breast cancer and delivery of doxorubicin (Tang et al., 2020), and the use of bitter melon EVs loaded with 5‐fluorouracil targeted against ocular surface squamous cell carcinoma (Yang et al., 2021). These vesicle‐mediated drug delivery approaches can replace the use of oil extracts with side effects resulting from low targeting while EVs facilitate various modes of delivery (such as intravenous delivery) owing to their lipid bilayer membrane protecting cargo in vivo. Exploring the profile of BasEVs can reveal potent biologicals that trigger apoptosis in cancer cells, ultimately, aiding in the isolation of other potent sub‐species in the basil plant genus or other traditional medicinal plants. Overall, our results here show that BasEVs can be used as a therapeutic agent and drug delivery platform for the treatment of pancreatic cancer.

## 4 CONCLUSION

To the best of our knowledge, this study is the first to report the isolation and characterization of EVs from the Basil plant. We studied its biological effects in pancreatic cancer and found that Basil plant EVs have intrinsic anti‐cancer properties. Based on our data, EVs of various other species of basil plant can be further explored for their potency in inhibiting cancer and are a good therapeutic candidate for in‐depth study in the pharmaceutical and nutraceutical sectors. With extracellular vesicle isolation technologies evolving in sophistication, achieving a high yield of EVs from largely abundant and fairly identical plants is feasible. Scale‐up of plant EVs as a therapeutic agent or a drug carrier is viable and addresses the challenges of low output, batch‐to‐batch variation, and undesired immunogenic profiles seen from the use of mammalian EVs. Further studies exploring the proteomic and genomic profiles of plant EVs can identify specific anti‐cancer biologicals. We show basil plant EVs possess the potency to trigger apoptosis in pancreatic cancer cells, which can be applied to enhance drug delivery.

## 5 MATERIAL AND METHODS

### 5.1 Plant material

Sweet Basil of the species *Osmium Basilica* of the mint family of *Lamiacaea* was used for their EVs in this study. Hydroponically grown basil plants were purchased from local market supplier (Herbanfarms, Cheyney, USA) and used within a day for EV isolation purposes.

### 5.2 Apoplast washing fluid collection

Apoplast washing fluid (AWF) was modified from previous studies (Huang et al., 2021). The proximal part of the leaves was removed, and distal zones of leaves were collected from plants. Leaves were carefully placed in a 2‐liter conical flask with a vacuum outlet and filled with infiltration buffer (20 mmol/L L2‐[N‐morpholino] ethane sulfonic acid (MES) hydrate, 2 mmol/L CaCl2, 0.1 mol/L NaCl, pH 6.0). A vacuum was gently applied until leaves turned dark with infiltration of the fluid (2–3 repetitions were performed as needed). Infiltrated leaves were gently wiped with paper towels to remove excess fluid. Initially, the weight of leaves before and after infiltration was measured to record the fluid infiltration amount. Dark infiltrated basil leaves were carefully placed in 50 mL conical plastic tubes with help of 20 mL syringes with apex of leaves facing upwards. The plastic tubes were centrifuged at 2000 g for 10 min at 4°C to collect the AWF.

### 5.3 Differential centrifugation to collect BasEVs

Basil AWF collected was used in isolation of BasEVs. AWF was filtered through a 0.45 μm filter and the filtrate was centrifuged at 10,000×*g* for 30 min at 4°C. Pellet was discarded, and the supernatant was centrifuged at 100,000×*g* for 60 min at 4°C. The supernatant was discarded, and the pellet resuspended in the infiltration buffer was centrifuged again at 100,000×*g* for 60 min at 4°C. Later, BasEV pellets were resuspended and collected in PBS or infiltration buffer for further experiments.

### 5.4 Characterization of BasEVs

BasEVs size was measured using Nanoparticle Tracking Analysis (NTA) (Zetaview, Particle Metrix). Basil EVs were diluted in PBS and loaded into the NTA device with a sensitivity set to 80% and a size threshold maximum set at 2000 nm equivalent parameters. BasEV size was plotted against the concentration of EVs/mL. TEM of BasEVs were performed as a service from Penn Electron Microscopy Resource Lab. BasEVs were stained with DiI (Tetramethylindocarbocyanine perchlorate) dye (Invitrogen) by mixing 0.1 μL of DiI in 20 μL BasEVs in PBS (3.5 × 10^10^ EVs/mL) and incubated for 5 min at room temperature. Later, BasEVs were run against Zeba Spin Desalting Columns or 7k filter to remove excess DiI dye (three runs). Dye‐stained BasEVs were visualized using a fluorescent microscope (Olympus IX83).

### 5.5 Western blot analysis

Protein analysis was performed on BasEVs using western blot technique. Briefly, 20 μL of 300 μg/mL BasEVs along with control mammalian cell (A431 lung cancer cell) EVs were spun down and resulting pellets treated with 1% Triton X‐100 for 30 min at room temperature (Protease inhibitor cocktail (PIC) added during lysis). After lysis, the lysate was treated with NuPAGE LDS sampling buffer and loaded into a 10%−12% pre‐casted gel (NuPAGE, Invitrogen, total volume of 20 μL per well). Electrophoresis was run at 50 V for 5 min and 150 V for 60 min followed by semi‐dry transfer to PVDF membrane (Invitrogen). Membrane was blocked with 5% dry skim milk for 1 h at room temperature followed by incubation with primary Heat shock 70 protein monoclonal antibody (MA3‐006, Invitrogen, 1:500) overnight at 4°C. Next day membrane was washed and incubated with secondary horseradish peroxidase (HRP) conjugated antibody followed by GAPDH HRP antibodies for 1 h at room temperature for both antibodies.

Immobilon Forte Western HRP Substrate (Millipore, #WBLUF0100) was applied on the membrane for chemiluminescence detection of protein.

For the Bax protein expression study, various treatment groups of cells were lysed using 1X Radioimmunoprecipitation assay (RIPA) buffer added with PIC. The cell lysate was processed similarly to the EV protein, where lysate was treated with NuPAGE LDS sampling buffer and loaded into 10%−12% pre‐casted gel. After running the gel, the membrane was blocked and then incubated with primary anti‐Bax antibody (Clone 6A7, EMD Millipore) overnight at 4°C. The membrane was then washed and incubated with HRP‐conjugated antibodies against Bax and GAPDH protein. Later, membranes were incubated with chemical substrate and imaged for chemiluminescence signal of proteins.

### 5.6 Basil plant EV uptake in cells

Similar to the characterization of basEV by staining with DiI, we stained the BasEVs with DiI and removed excess DiI using Zeba spin desalting columns (Thermo Fischer). MIA PaCa‐2 cells were seeded at 30,000 cells/well in an 8‐well chamber apparatus (CELLTREAT). After overnight culture, various concentrations of BasEVs (40, 80, and 160 μg/mL) were mixed in complete DMEM growth media (10% FBS and 1% Penicillin‐Streptomycin) and given to the cells. Following 4 h incubation, cells were washed 3X PBS and stained with Hoechst stain for nucleus visualization. Live cells after staining were visualized immediately under a fluorescent microscope (Olympus IX83) and z‐stack images were recorded for co‐localization purposes.

### 5.7 Cell viability studies

Cell viability of BasEV‐treated pancreatic cells was performed via two different assays. Cell viability quantification was performed via MTS assay (Promega). MIA PaCa‐2 pancreatic cancer cells or HEK293T cells were seeded in 48‐well plates at confluence and grown overnight at 37°C and 5% CO2 in DMEM complete media (GIBCO). The next day, BasEVs at various concentrations (40, 80, 160 μg/mL) were given to the cells suspended in media along with no treatment and basil oil as controls. Basil oil purchased from NOW essential oil company (Bloomingdale's, Illinois) was used as a positive control at 1.7 (%v/v). 100% ethanol was used as a positive control where 30 min before the end of treatment, one group of untreated cells media was replaced with ethanol. After 48 h, MTS reagent (Promega) was added to all groups and incubated for 40 min. Absorbance at 490 nm was recorded from all the wells and plotted, normalizing against the untreated control. Similar to the MTS assay, after incubation with BasEVs and controls, cells were stained with a LIVE/DEAD staining kit (Invitrogen) for 15 min, and cell were washed and imaged using a fluorescent microscope (ECHO).

Similarly, to dose dependent BasEV inhibition studies, we compared supernatant from AWF and the BasEVs inhibitory properties. We used the supernatants isolated from last two steps of ultracentrifugation at 100,000×*g* labelled as P‐100 (second to last step) and P2‐100 (last step). BasEVs and supernatant protein concentration was normalized, and equal concentration of protein fractions were given to MIA PaCa‐2 cells cultured overnight in 96‐well plates. After 24 h, MTS assay was performed to assess the cell cytotoxicity from the supernatant as well as BasEVs.

For combinatorial studies pertaining to BasEV and gemcitabine (Fischer Scientific), a gemcitabine concentration of 10 μM and BasEVs of 160 μg/mL was used. Various groups assessed included BasEVs, gemcitabine, BasEV and gemcitabine (co‐administered), BasEV treatment followed by gemcitabine treatment (after 4 h), and untreated control.

### 5.8 Caspase assay

MIA PaCa‐2 cells were seeded in a 96‐well plate overnight at 10,000 cells/well. The next day, BasEVs (160 μg/mL) and basil oil treatments were given to the cells. After 48 h, Caspase‐Glo 3/7 (Promega) reagent mix was added to the cells and incubated for 30 min. Luminescence from various groups were recorded using a plate reader (Glomax system, Promega).

### 5.9 RT‐PCR study of apoptotic genes

MIA PaCa‐2 pancreatic cancer cells were seeded into 48‐well plates and grown overnight. The next day, BasEVs were given to the cells at a concentration of 160 μg/mL. After 48 h, cells were washed and harvested for RNA using TRIzol reagent (Invitrogen). Extracted RNA was treated with DNase TURBO (ThermoFisher) and reverse transcribed to cDNA using iScript cDNA Synthesis Kit (Bio‐Rad). The cDNA product was then analysed via RT‐PCR using the SsoAdvanced Universal SYBR Green Supermix (Bio‐Rad) on the StepOnePlus Real‐Time PCR System (ThermoFisher) for the following apoptotic and housekeeping genes: Bax, Bcl2, Caspase3, Survivin, and GAPDH. Primers for the genes are available in Table S1.

### 5.10 Clonogenic survival assay

To assess the BasEVs effects on the clonogenic potential of pancreatic cancer cell lines, a clonogenic survival assay was performed in a 12‐well plate. MIAPaCa‐1 cells were seeded at 5000 cells/well and allowed to attach overnight. The next day, the cells were treated with 160 μg/mL of BasEVs or IC50 of basil oil. After 1 week, cells were washed and stained using crystal violet to count colonies using the method previously described (Yang et al., 2012). Briefly, cells after treatment and incubation for a week, were washed and fixed with acetic acid/methanol in a 1:7 ratio for 5 min at room temperature. Later, cells were washed and fixed with 0.5% crystal violet for 2 h at room temperature. After crystal violet staining, cells are washed and detached using media with 10% FBS. Finally, the dishes were rinsed in tap water and air‐dried for 1–2 days before counting colonies. The below formulas are used to calculate the plating efficiencies (PE) and surviving fraction (SF).

$$PE=\frac{no.\;of\;colonies\;formed}{no.\;of\;cells\;seeded}x100$$

$$SF=\frac{no.\;of\;colonies\;formed\;after\;treatment}{no.\;of\;cells\;seeded}x\;PE$$

### 5.11 Mass spectrometric analysis of proteins

BasEV pellets were digested using trypsin to extract peptides as previously described (Chaya et al., 2023). Tandem mass spectra were extracted from peptides of BasEV pellet and supernatant before collecting the pellets labelled P‐100. All MS/MS samples were analysed using MaxQuant (Max Planck Institute of Biochemistry, Martinsried, Germany; version 1.6.14.0). MaxQuant was set up to search the U_Ocimum_2023_09_11 database assuming the digestion enzyme stricttrypsin.

MaxQuant was searched with a fragment ion mass tolerance of 40 PPM and a parent ion tolerance of 20 PPM. Carbamidomethyl of cysteine was specified in MaxQuant as a fixed modification. Oxidation of methionine and acetyl of the n‐terminus were specified in MaxQuant as variable modifications. Scaffold (version Scaffold_5.3.0, Proteome Software Inc., Portland, OR) was used to validate MS/MS based peptide and protein identifications. Peptide identifications were accepted if they could be established at greater than 81.0% probability to achieve an FDR less than 1.0%. Peptide probabilities from MaxQuant were assigned by the Peptide Prophet algorithm with Scaffold delta‐mass correction (Keller et al., 2002). Peptide probabilities from MaxQuant data of samples were assigned by the Percolator posterior error probability calculation (Käll et al., 2008). Protein identifications were accepted if they could be established at greater than 6.0% probability to achieve an FDR less than 5.0% and contained at least two identified peptides.

Protein probabilities were assigned by the Protein Prophet algorithm. Proteins that contained similar peptides and could not be differentiated based on MS/MS analysis alone were grouped to satisfy the principles of parsimony.

### 5.12 Statistical analysis

GraphPad Prism8 (GraphPad Software Inc) was used to perform all statistical analysis. One‐way ANOVA with Tukey's multiple comparison tests was done for data analysis. Triplicate samples were used for all the studies if not specified. Standard error mean was employed for plotting data.

## AUTHOR CONTRIBUTIONS

**Uday Chintapula**: Data curation; formal analysis; investigation; software; visualization; writing—original draft; writing—review and editing. **Daniel Oh**: Data curation. **Cristina Perez**: Data curation. **Sachin Davis**: Data curation. **Jina Ko**: Conceptualization; resources; supervision; writing—review and editing.

## CONFLICT OF INTEREST STATEMENT

The authors declare no competing financial interest.

## Supporting information

Supporting Information

## Data Availability

The data that support the findings of this study are available from the corresponding author upon reasonable request.
