# Supplementary material for: Anti‐cancer bioactivity of sweet basil leaf derived extracellular vesicles on pancreatic cancer cells
Source: J Extracell Biol. 2024 Feb 1;3(2):e142. doi: 10.1002/jex2.142 (PMC11080924; doi:10.1002/jex2.142)
Supplement: Supplementary file 1 — Supporting Information [file JEX2-3-e142-s001.docx]

**Anti-cancer bioactivity of Basil plant extracellular vesicles on pancreatic tumor cell lines**

Uday Chintapula, Oh Danny, Cristina Perez, Sachin Davis, Jina Ko

**Table S1.** Yield of BasEVs for various rounds of isolation

| **Weight of leaves used (grams)** | **EV protein isolated (µg/ mL)** | **NTA size/count (nm)/(EVs/mL)** |
| --- | --- | --- |
| 20 | 22 | 152 ± 75/ 4.7 x 108 |
| 99 | 120 | 129±27 / 3.5 x 108 |
| 200 | 320 | 111 ± 11/ n/a |
| 600 | 500 | 178 ± 81/ 3.3 x 1010 |

**Table S2.** Primer sequences used for RT-PCR experiments

| **Genes** | **RT-PCR Primer sequences** |
| --- | --- |
| Bax | Forward-CAAGGCCCTGTGCACTAAAGT |
|  | Reverse -AAGTAGGAGAGGAGGCCTTCC |
| Bcl2 | Forward-GGAGAAATCAAACAGAGGTCGC |
|  | Reverse -CGTCAACAGGGAGATGTCACC |
| Caspase-3 | Forward-GGAGCAGCTTTGTGTGTGTG |
|  | Reverse -TCCAGGAATAGTAACCAGGTGC |
| Survivin | Forward-GTTGCGCTTTCCTTTCTGTC |
|  | Reverse -TCTCCGCAGTTTCCTCAAAT |
| GAPDH | Forward-GGAAGGTGAAGGTCGGAGTCA |
|  | Reverse -GTCATTGATGGCAACAATATCCACT |


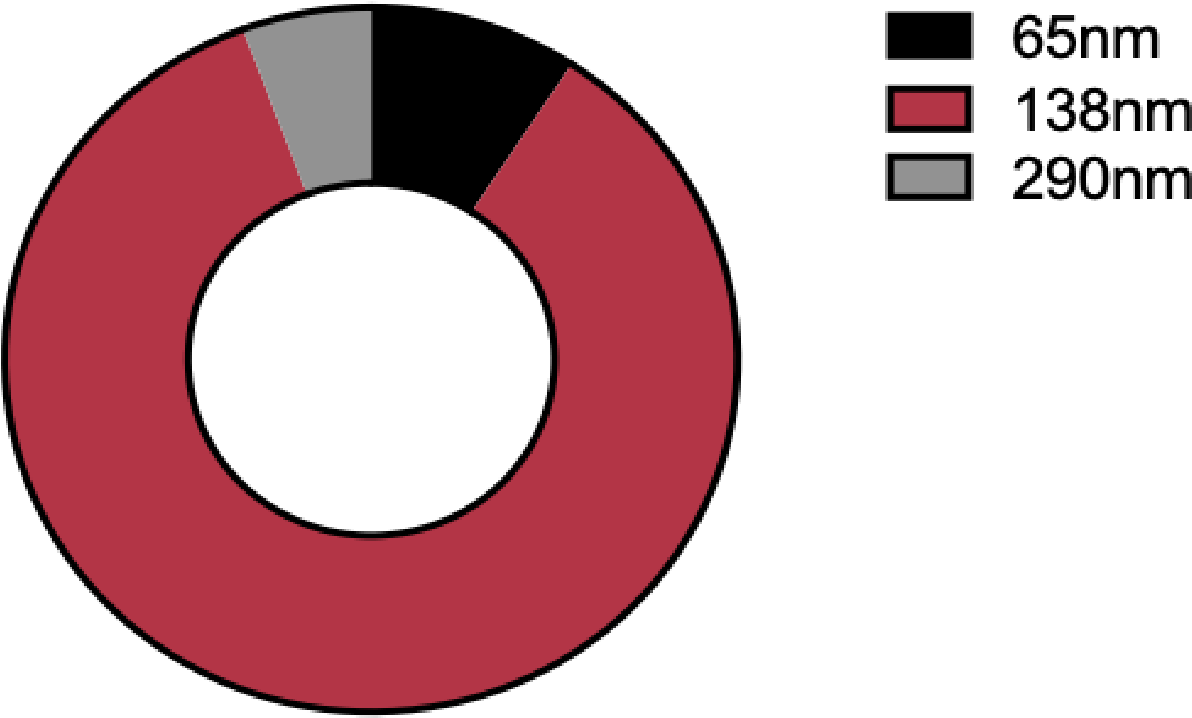


**Figure S1**. Distribution of various diameters of Basil EVs as measured by Nanoparticle Tracking

**70**

**KDa**

**HSP70**

**37**

**KDa**

**GAPDH**


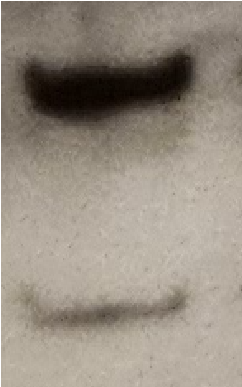

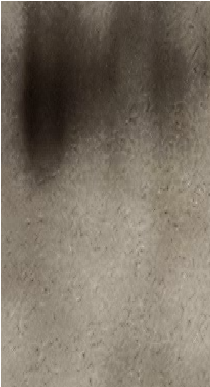


**Mammalian**

**EVs**

**Basil**

**EVs**

**Figure S2.** **Western blot analysis of BasEVs.** BasEVs and mammalian EVs were assessed for Heat shock protein 70 and GAPDH proteins. Compared to mammalian cell (A431 lung cancer cells) EVs, BasEV signal for HSP70 is weak yet still visible which maybe due to less affinity of the antibody. BasEV do not show any GAPDH proteins


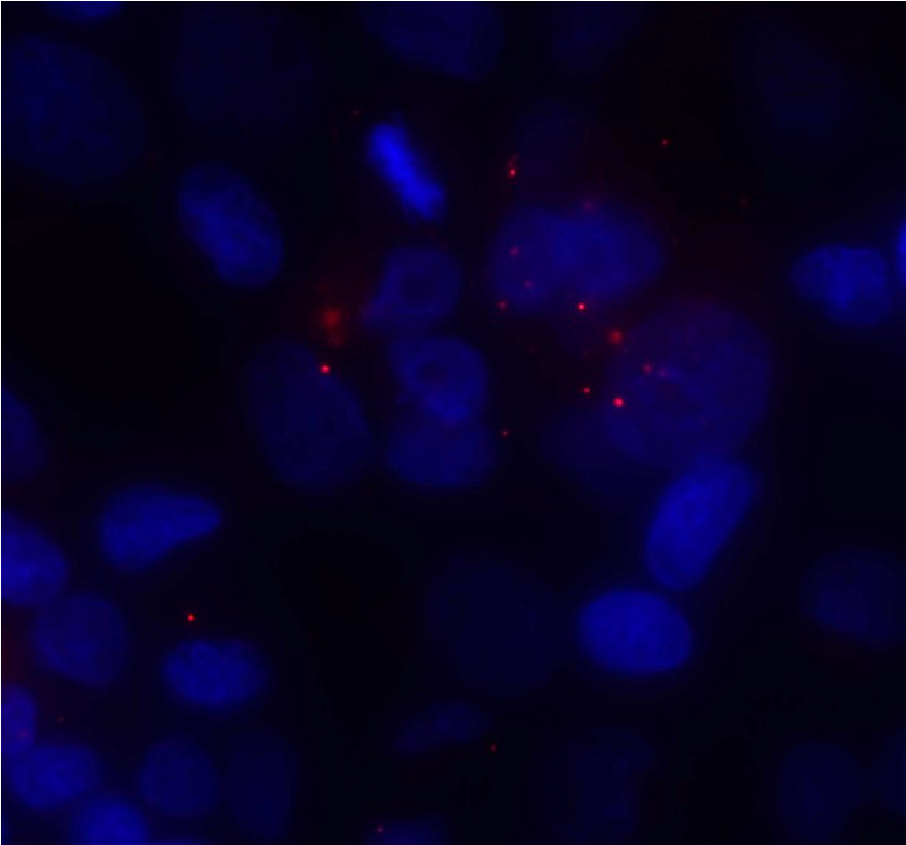

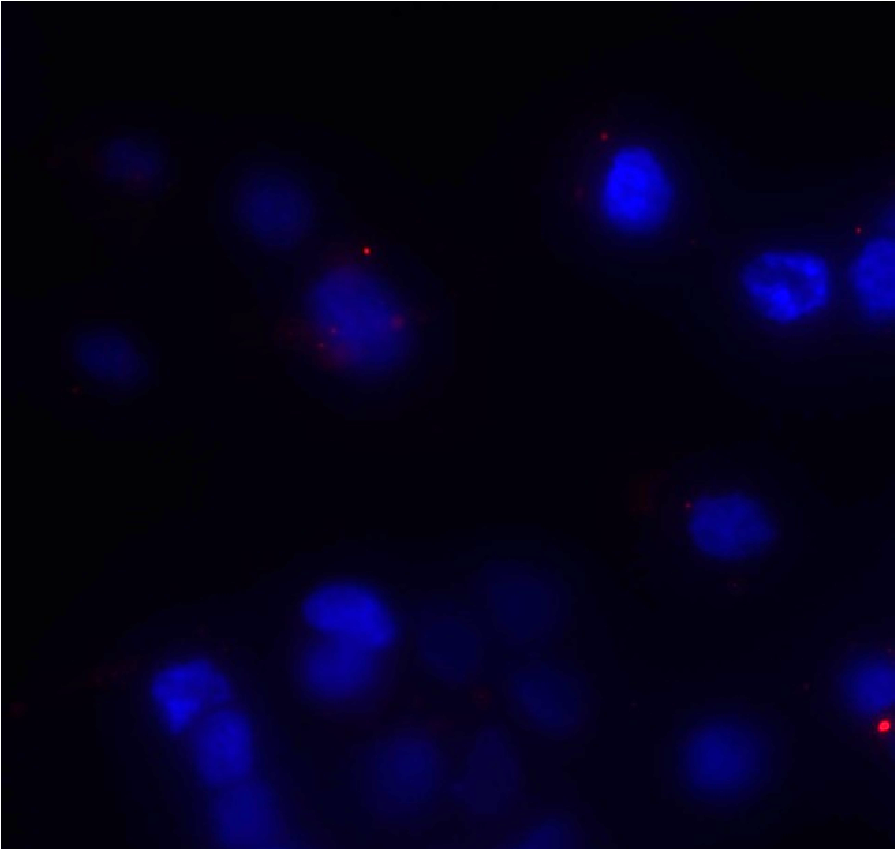


**b**

**a**

**Figure S3**. Basil EV uptake in pancreatic cell lines a. BasEV uptake in AsPC-1 cells. b. BasEVs uptake in PanC1 pancreatic cells. Nucleus is stained with Hoechst and BasEVs stained with DiI dye (shown with green arrows)

**Video S1**. Z-stack video of BasEV uptake in MIA PaCa-2 cell line (BasEVs in TRITC channel (red)):

[BasEV uptake in pancrea0c cells](https://drive.google.com/file/d/10P3LMBeEteLcx7DORS9yNNOr4cg1FIxM/view?usp=drive_link)

**Figure S4. BasEVs effects of cell growth in HEK293T cells.**

MTS assay results showing a dose-

dependent activity in cell viability of HEK293T cells after BasEV treatment.


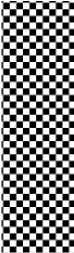

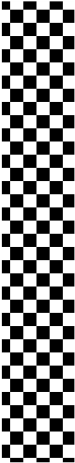

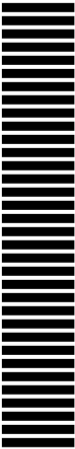

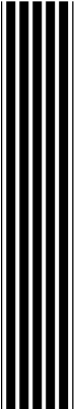


untreated

Basil-oil

Basil EVs(40)

Basil EVs(80)

Basil EVs(160)

Ethanol

**0**

**50**

**100**

**150**

Cell viability

(Normailized to untreated)

✱

✱

ns


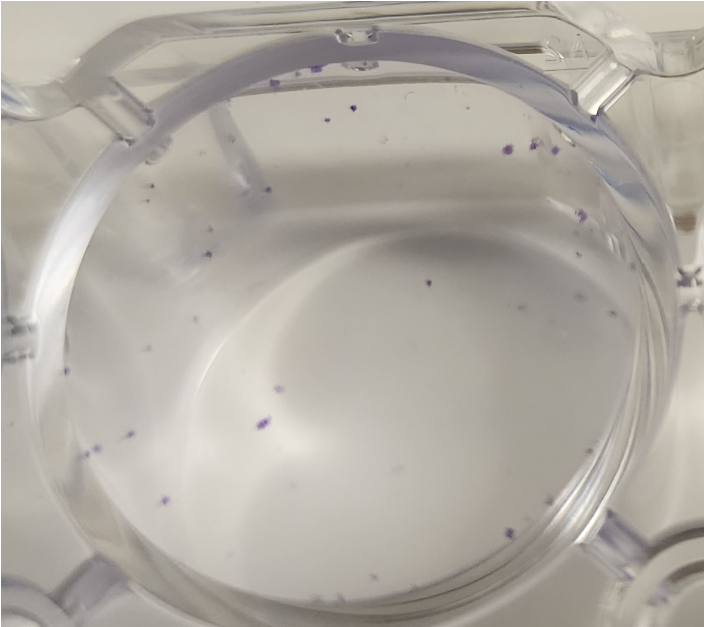

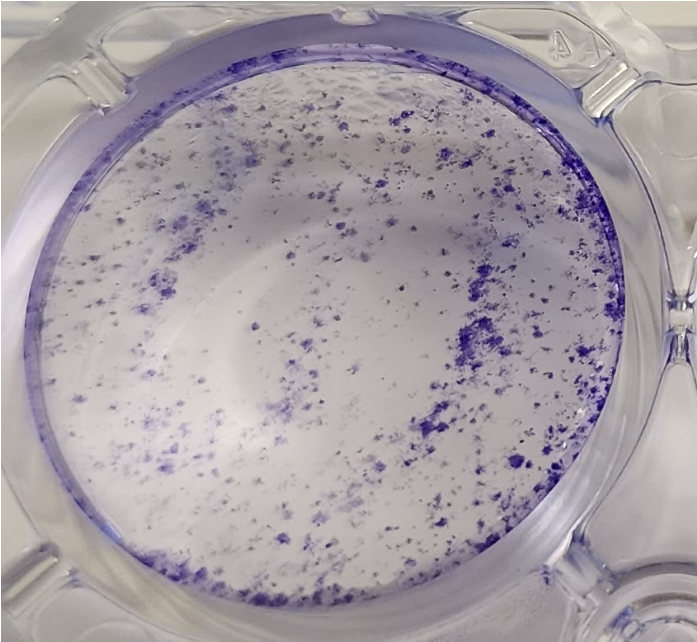

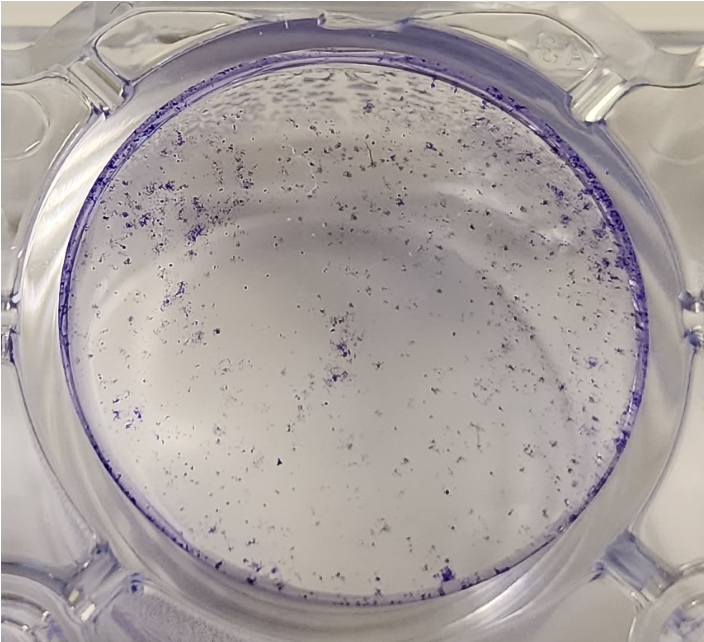


Untreated

Basil EVs

Basil Oil

**b**

**a**

**c**

Basil EVs

Basil oil

0.000

0.002

0.004

0.006

0.008

Survival Fraction

untreated

Basil EVs

Basil oil

0

20

40

60

80

No. of Colonies formed

✱

✱✱✱

✱✱

**Figure S5. Clonogenic Survival Assay** **a.** Survival fraction of BasEVs and Basil Oil Normalized to the control. **b.** No of colonies formed after treatment with BasEVs and Basil Oil **c.**Well plate images showing the colony formation in untreated, Basil EV and Basil Oil treatment groups

Untreated

Gem

BasEVs

BasEVs+Gem

PT-BasEVs+Gem

**0**

**50**

**100**

**150**

Cell viability

(Normailized to untreated)

✱✱

✱

✱

✱

**Figure S6. BasEVs and Gemcitabine combinatorial treatment for 12hrs.** MTS assay results showing a combinatorial effects of BasEVs and Gemcitabine (IC50 of 10 µM in MIA PaCa-2 cells).

**Figure S7.**

**Caspase Activity Assay.**

Caspase activity was recorded in BasEV and Basil Oil treated cells

**BasEVs**

**Basil oil**

**0.0**

**0.5**

**1.0**

**1.5**

**Caspase-3/7 Luminescence**

**)**

**(**

**Normalized to control**

with data normalized to untreated control **Figure S8.** **Inhibitory effects of soluble components and EVs from sweet basil Apoplastic Washing Fluid**. P-100 (second to last ultracentrifugation spin) supernatant, P2-100 ( last ultracentrifugation spin) supernatant and BasEVs (from final pellet) were given to MIA PaCa-2 in a normalized protein concentrations (160 µg/mL) to study their cytotoxicity.

Untreated

BasEVs

P-100

P2-100

**0**

**50**

**100**

**150**

Cell viability

(Normailized to untreated)

✱


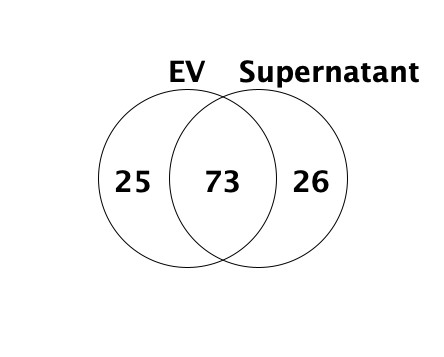


**Figure S9.**

Overview of proteins identified in BasEV (EV) and apoplastic washing

fluid (Supernatant)

**Table S3. BasEV Proteins (**✔ **= presence detected)-1**

| **Protein list** | **Detected**  **in**  **BasEVs** | **Detected in supernatant**  **(Apoplastic washing fluid)** | **Functional relevance** |
| --- | --- | --- | --- |
| Ribulose bisphosphate carboxylase | ✔ | - | Carbon fixation |
| Ribulose-1,5-bisphosphate carboxylase/ oxygenase | ✔ | ✔ | Carbon fixation |
| Photosystem II CP47 reaction center protein: oxygenic photosynthesis | ✔ | - | Oxygenic photosynthesis |
| Photosystem I P700 chlorophyll apoprotein | ✔ | - | - |
| Cytochrome f | ✔ | - | Oxygenic photosynthesis |
| ATP synthase | ✔ | - | Oxygenic photosynthesis |
| NADPH-quinone oxireductase | ✔ | - | - |
| Eugenol O-methyltransferase | ✔ | - | Enzyme to synthesize methyleugenol - a potent anti-cancer agent |
| Glyceraldehyde-3-phosphate dehydrogenase: glycolysis | ✔ | ✔ | Glycolysis |
| 30S ribosomal protein | - | ✔ | - |
| Eugenol synthase | ✔ | ✔ | Enzyme involved in eugenol synthesis |
| 50S ribosomal protein | ✔ | ✔ | - |
| Cobalamine-independent methionine synthase | ✔ | ✔ | Plant specific enzyme involved in B12 pathway |

| Geraniol dehydrogenase | ✔ | ✔ | - |
| --- | --- | --- | --- |
| Hydroxyphenylpyruvate reductase | - | ✔ | Glycolysis |
| Lectin 2 | - | ✔ | - |
| Protein TIC 214 | - | ✔ | - |
| Ferredoxin NADP+ reductase | - | ✔ | - |
| Caffeic acid 3-O-methyltransferase | - | ✔ | - |
